# Institutional Trends in Penicillin Allergy: A New Era of Active Penicillin Allergy Delabeling

**DOI:** 10.1111/cea.70320

**Published:** 2026-04-21

**Authors:** Anna Brameli, Allison B. McCoy, Elizabeth McNeer, Leena Choi, Joanna L. Stollings, Grace Koo, Allison E. Norton, Milner Staub, George E. Nelson, James W. Antoon, Sophie E. Katz, Ritu Banerjee, Adam Wright, Elizabeth J. Phillips, Cosby A. Stone

**Affiliations:** 1 Division of Allergy, Pulmonary and Critical Care Medicine, Department of Medicine, Vanderbilt University Medical Center, Nashville, Tennessee, USA; 2 Division of Infectious Diseases, Department of Pediatrics, Monroe Carell Children’s Hospital, Vanderbilt University Medical Center, Nashville, Tennessee, USA; 3 Department of Biomedical Informatics, Vanderbilt University Medical Center, Nashville, Tennessee, USA; 4 Department of Biostatistics, Vanderbilt University Medical Center, Nashville, Tennessee, USA; 5 Department of Pharmacy, Vanderbilt University Medical Center, Nashville, Tennessee, USA; 6 Division of Allergy, Immunology, and Pulmonary Medicine, Department of Pediatrics, Monroe Carell Jr. Children’s Hospital at Vanderbilt, Nashville, Tennessee, USA; 7 Division of Infectious Diseases, Department of Medicine, Vanderbilt University Medical Center, Nashville, Tennessee, USA; 8 Division of Hospital Medicine, Department of Pediatrics, Monroe Carell Children’s Hospital, Vanderbilt University Medical Center, Nashville, Tennessee, USA

## To the Editor,

Penicillin allergy labels (PALs) have a reported worldwide pooled prevalence of 9.4% (ranging from 5% to 15%), and pose a major public health challenge [1]. PALs are associated with broader-spectrum antibiotics, longer hospital stays, treatment comorbidities and increased healthcare costs [2]. However, true penicillin allergy is rare and most reported reactions are ‘low-risk’, which carry only a 0.5%–2% chance of a rash recurrence when rechallenged [3]. The vast majority of these labels originate in early childhood with approximately 75% of PALs acquired by the age of 3 years [4], often when viral or secondary bacterial infections trigger non-specific rashes inaccurately diagnosed as drug allergies. Historically, paediatricians and internists recommended life-long avoidance, driving current high PAL prevalence.

Recent advancements in PAL management advocate for risk-stratification and direct oral challenges for low-risk patients [5]. At Vanderbilt University Medical Center (VUMC) and Monroe Carell Jr. Children’s Hospital, active multi-setting delabelling programmes were implemented starting in 2014. These targeted interventions started in the outpatient adult and paediatric drug allergy clinics but widened to include an inpatient adult care [6] and the children’s hospital, and the obstetric setting. In each setting, dedicated, trained personnel implemented a risk-stratification algorithm to identify low-risk patients for direct oral challenges. Until now, institutional trends in PAL prevalence following such interventions were unexplored. We aimed to evaluate PAL prevalence, including additions and deletions, along with antibiotic prescribing trends across our healthcare system from 2018 to 2024.

Briefly, we retrospectively analysed electronic health record (EHR) data to determine annual PAL prevalence and antibiotic prescriptions across 205,661 paediatric patients (0–17 years) (range 169,510–239,125), 436,808 patients 18–64 (range 372,536–477,006) and 136,510 patients aged 65+ (range 108,832–162,121). We utilised linear regression and spline models to assess time trends, tracking PAL addition and deletion during healthcare encounters. We also identified all patients’ prescriptions for amoxicillin, cephalexin, azithromycin or clindamycin.

**FIGURE 1 |:**
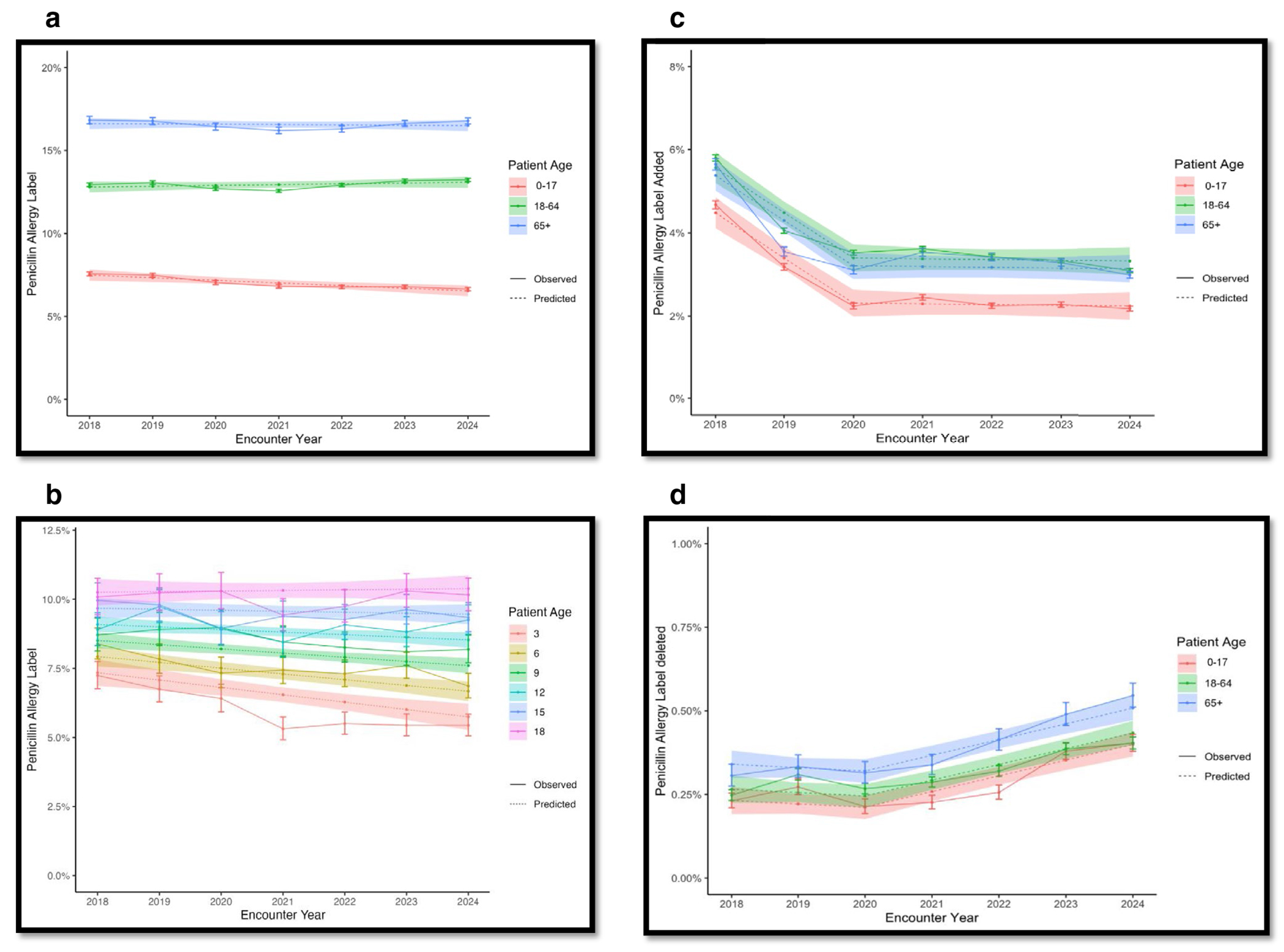
(a) Prevalence of penicillin allergy labels in patients in all age groups. (b) Prevalence of penicillin allergy labels in paediatric patients by age group. (c) Penicillin allergy labels added by age group. (d) Penicillin allergy labels delabelled by age group. *These plots show the predicted values (dotted lines) and 95% confidence intervals (shaded bands) from the regression analysis along with the observed values (solid lines) and pointwise 95% confidence intervals (vertical bars).

Our main results show that between 2018 and 2024, paediatric PAL prevalence demonstrated a significant and sustained decrease from 7.57% in 2018 to 6.65% in 2024. The regression analysis showed a significant interaction between time and age group (interaction term *p*-value = 0.013) and the paediatric group PAL decreased by approximately 0.16% each year. This declining trend was unique to the paediatric cohort; conversely, PAL prevalence slightly increased in adults (12.94% to 13.24%) and remained stable in older adults (~16.8%) over the same period (Figure 1a).

A deeper examination of the paediatric cohort revealed that the observed decline in PAL prevalence was most pronounced among the youngest children.

Regression modelling showed that the trend in PALs over time differed significantly by age (interaction term *p*-value = 0.011) with a 0.02 increase in the time trend per one year of age, with an estimated decrease of 0.27% per year for 3-year-olds. In contrast, 18-year-olds experienced a slight estimated increase of 0.04% per year (Figure 1b).

We also observed a decrease in the addition of new PALs, with a particularly pronounced decrease from 2018 to 2020 (regression analysis estimated a decrease of 1.08%), and a flatter trend from 2020 to 2024 (0.02% decrease annually) (Figure 1c). However, this finding may also be attributed to a transition to a new EHR system (Epic), during which an entry of preexisting allergy labels occurred in a large bolus and later removed from the medical record.

Across all age cohorts, the rate of PAL deletion (delabelling) increased significantly. Prior to 2020, deletion rates were relatively flat, but rose by approximately 0.05% per year afterwards, reflecting the successful expansion of our delabelling initiatives (Figure 1d).

To evaluate our stewardship priorities in a global context, we aligned our data with the World Health Organization (WHO) AWaRe (Access, Watch, Reserve) classification system [7]. This framework aims to curb antimicrobial resistance, targeting at least 60% of consumption from first-line ‘Access’ group antibiotics. Guided by these targets and our institutional priorities, we analysed prescription trends for specific targeted antibiotics: amoxicillin and cephalexin (preferred ‘Access’ beta-lactams), alongside azithromycin (a ‘Watch’ antibiotic), and clindamycin (an alternative the AWaRe recommends limiting).

In all age groups amoxicillin use, which had decreased prior to 2020, rebounded and increased significantly post-2020 alongside cephalexin. Meanwhile, clindamycin use had a decreasing trend of 0.05% (95% CI 0.03%–0.06%, *p* < 0.001) per year. These prescribing patterns suggest that the observed reduction in paediatric PALs is not an artefact of decreased beta-lactam utilisation; rather, it might reflect a genuine progress driven by active delabelling, heightened clinical awareness among paediatricians and robust antimicrobial stewardship that actively encourages WHO-preferred first-line beta-lactam usage over alternatives.

Our study has several limitations including its retrospective nature, which cannot establish causality or fully explain the factors driving trends in PAL labelling or antibiotic prescribing, as well as the ability to get data extending only back to 2018, when our institution transitioned EHR systems, limiting the historical comparisons. We also cannot determine whether PALs reflect preexisting labels or new reactions. In addition, the COVID-19 pandemic affected infection rates and prescribing patterns, complicating trend interpretation.

Despite these limitations, we observed a meaningful 1% reduction in paediatric PAL prevalence and increased delabelling across all ages.

Further reductions in PAL burden at institutional scale are anticipated to bring more effective care delivery, better antimicrobial stewardship, improved treatment outcomes and decreased costs [8, 9].

While more research is clearly needed, we hypothesise that a key transition point may have occurred, wherein an active delabelling strategy played a role, but also a more accurate assessment of early childhood reactions began to prevent unnecessary PALs from being assigned. Such effects may shift PAL population prevalence downward for the next generation. In conclusion, our data demonstrate that implementing active, multi-setting delabelling programmes can successfully reduce an institution’s long-term PAL burden and promote optimal first-line beta-lactam utilisation.

## Data Availability

The data that support the findings of this study are available from Vanderbilt University Medical Center but restrictions apply to the availability of these data, which were used under IRB approval for the current study, and so are not publicly available. Data are however available from the authors upon reasonable request and with the permission of Vanderbilt University Medical Center.

## Summary

- Paediatric penicillin allergy prevalence declined from 7.57% to 6.65% (2018–2024), especially in youngest children.
- An overall decrease in new PAL and an increase in institutional penicillin allergy delabelling was observed.
